# Psychometric properties and validation of short health literacy screening (SHLS) to identify low health literacy

**DOI:** 10.34172/hpp.025.44250

**Published:** 2025-12-30

**Authors:** Sulmaz Ghahramani, Marzieh Rahimi, Majid Khadem-Rezaiyan, Mina Zarei, Mohammad Sayari, Ali Ardekani, Kamran Bagheri Lankarani

**Affiliations:** ^1^Health Policy Research Center, Institute of Health, Shiraz University of Medical Sciences, Shiraz, Iran; ^2^Endoscopic and Minimally Invasive Surgery Research Center, Mashhad University of Medical Sciences, Mashhad, Iran; ^3^Medical Sciences Education Research Center, Mashhad University of Medical Sciences, Mashhad, Iran; ^4^Department of Community Medicine, Faculty of Medicine, Mashhad University of Medical Sciences, Mashhad, Iran; ^5^Department of Medicinal Chemistry, School of Pharmacy, Shiraz University of Medical Science, Shiraz, Iran; ^6^Department of Mathematical Sciences and Research Methods Centre, Durham University, Durham, United Kingdom

**Keywords:** Adult, Health literacy, Iran, Public health, Validation study

## Abstract

**Background:**

The purpose of this study was to evaluate the psychometric properties and performance of the short health literacy screening (SHLS) to identify people with low health literacy among a set of Iranian adults in Mashhad and Shiraz.

**Methods:**

In this cross-sectional study, participants were 18-65 years old Iranian adults randomly selected from two major cities of Iran, encompassing inpatients, outpatients, and healthy individuals. They completed the demographic, SHLS, and Health Literacy for Iranian Adults (HELIA) questionnaires. Psychometric properties of SHLS were evaluated. We investigated the performance of screening questions for detecting inadequate and limited health literacy based on HELIA as a comparison standard.

**Results:**

SHLS had acceptable psychometric properties (Content validity index of 0.92, 0.78, 0.85, and 0.85, Spearman-Brown correlation coefficient of 0.827, 0.928, 0.581, and 0.712 for four questions, and Cronbach’s alpha of 0.713). Of 547 participants, 339 (62%) were female, and 415 (75.9%) were between 18 and 45 years old. The prevalence of inadequate and limited health literacy based on HELIA was 56 (10.2%) and 192 (35.1%), respectively. The question "How well do you understand the medical prescriptions your doctor told you?" (Comprehension of Prescriptions) showed a higher area under the receiver operating characteristic (AUROC) of 0.690 (0.620-0.759) for inadequate, and 0.666 (0.619-0.713) for limited health literacy. The combination of "How confident are you filling out forms by yourself?" and "Comprehension of Prescriptions" showed a better AUROC of 0.705 (0.638-0.773) for inadequate health literacy. All questions together had a higher AUROC of 0.683 (0.636-0.729) for limited health literacy. The response "Quite a bit" or less confident for "Comprehension of Prescriptions" increased the odds of inadequate and limited health literacy by 1.45- and 1.53-fold, respectively.

**Conclusion:**

The question "Comprehension of Prescriptions" and the four-item SHLS could identify people with low health literacy.

## Introduction

 Health literacy, as a key determinant of health, is crucial for improving healthcare services and reducing health disparities.^1^ Health literacy is defined by the World Health Organization (WHO) as “cognitive and social skills, which determine the motivation and ability of individuals to gain access to understand and use information in ways which promote and maintain good health”.^2^ It is more than just reading and writing; it involves several abilities that enable people to interact more actively in their community and take a greater role in their own health-related actions and decision-making.^3^ Inadequate or low health literacy has become a global public health concern.^4^ Poor health literacy has been related to non-adherence to medications, higher rate of hospitalization, reduced use of preventive interventions, greater health care costs, higher mortality rates in the elderly, as well as worse overall health status. Low literacy also perpetuates existing disparities and follows a socioeconomic gradient.^5^ Along with the growing attention to health literacy at both national and international scales, there has been an increasing need to develop reliable and valid tools for the measurement of health literacy.^6^ The evaluation of health literacy in individuals seeking care, and even healthy people, may contribute to improvement of care delivery by healthcare facilities.^7^

 There are some validated tools to evaluate health literacy, including Rapid Estimate of Adult Literacy (REALM)^8^ and Short Test of Functional Health Literacy in Adults (STOFHLA).^9,10^ However, available instruments are either too time-consuming and could make patients feel embarrassed.^11^ In addition, the majority of standardized instruments were designed and developed for English-speaking individuals, potentially limiting their utility in multicultural nations. It has been recognized that numerous health literacy tools do not account for the cultural and contextual factors affecting health literacy.^12^ Recognizing patients’ cultural backgrounds and values helps to improve the validity of research tools. Surveys that are brief, simply worded, easy to understand, and presented by professionals who speak the local language will facilitate the trust and confidence of the target population.^12^ In busy clinical settings, short and easy-to-use screening tool could help physicians identify people with limited health literacy, who may need special support and communication methods. Large-scale research to comprehend the impacts of health literacy and the efficacy of interventions would also be more feasible with these screening tools.^13^ Brief health literacy screening questions were developed by Chew et al^14^ to assess perceived difficulties in understanding, reading, and reporting medical information. It provides a quick method for detecting patients with low health literacy, making it suitable for clinical use.

 Although this screening tool has been translated into several languages,^15-18^ no study has investigated its psychometric properties among the Iranian people. To our knowledge, there has been no implementation of a screening questionnaire for evaluating health literacy in Iran, which encompasses the majority of health literacy components and aligns with the values of Iranian culture. Considering the critical role of health literacy in public health, it is beneficial to screen health literacy levels among the Iranian population. This endeavor will serve as a valuable tool for evaluating whether the social and health ambitions of Iran aimed at sustainable health promotion are successfully met. Therefore, the aim or our study was to assess the psychometric properties and performance of Chew screening questions, along with a fourth question developing based on our context, as short health literacy screening (SHLS), to identify individuals with inadequate and limited health literacy based on Health Literacy for Iranian Adults (HELIA) questionnaire in a large sample of inpatients, outpatients and healthy individuals in two large cities in Iran.

## Methods

###  Study Population

 A cross-sectional study was conducted to investigate the psychometric properties of health literacy screening questions between April 2023 and September 2023. For a comprehensive evaluation of the health literacy of Iranian people, we conducted our research in two large cities in Iran: (1) Mashhad, (2) Shiraz. In each city, we collected our study participants from three types of people: (1) inpatients, (2) outpatients, and (3) healthy individuals.

- Inpatient individuals were collected from internal medicine and surgery departments of three types of hospitals: (1) public (governmental), (2) semi-private, and (3) private hospitals. Public hospitals were referral and governmental. Semi-private hospitals were non-referral, and part of their budget was funded by the government. Private hospitals were non-referral, and their budget was provided by the private sector. The required sample size from inpatients was obtained by the convenience sampling method on randomly selected days of the week.
- Outpatients were collected from two types of clinics (with different socio-economic status): (1) public (governmental), and (2) private clinics. Public clinics that were governmental and referral or non-referral (two types of public clinics were considered). Private clinics were non-referral, and their budget was provided by the private sector. The required sample size from outpatients was obtained by the convenience sampling method on randomly selected days of the week.
- Healthy people were selected from non-first-degree healthy companions of hospitalized patients who had no history of hospitalization in the last year. The required sample size from healthy individuals was obtained by the convenience sampling method on randomly selected days of the week, on the same days as the inpatients were collected.

We conducted this study to determine whether the diagnostic sensitivity of the SHLS for detecting low health literacy is at least acceptable. Based on conventional screening benchmarks—where values < 0.70 are commonly regarded as sub-optimal for screening tools and ≥ 0.80-0.85 as desirable—we set the null hypothesis at p₀ = 0.70 (minimum acceptable sensitivity) and the alternative at p₁ = 0.85 (anticipated sensitivity). With a two-sided alpha = 0.05 and 80% power, using Bujang and Adnan^19^ method, the required sample size was n = 82 for testing sensitivity against p₀. Because the target population comprised inpatients, outpatients, and healthy individuals, and we planned stratified analyses to ensure each subgroup met the same minimum operating characteristics, we applied n = 82 per stratum (total n = 246 per city). To maintain interpretability across subgroups, we aimed for approximately equal recruitment within each stratum. With two cities, the planned sample was n = 492, and allowing ~10% attrition/non-evaluable data, the final target was n = 540.

 To be eligible, participants had to be Iranian, able to speak Persian, and between 18 and 65 years old. Study participants who claimed to be illiterate were excluded from the study. In addition, we excluded individuals who were too ill to complete the questionnaire, had diminished decision-making capacity, severely impaired vision, hearing problems, overt psychiatric disorders, and severe cognitive impairments.

###  Study Protocol

 The study purpose was explained to all participants by trained research assistants, and they were informed that their responses would be anonymous. After obtaining informed consent, they completed the questionnaire, which included questions about demographic and socioeconomic characteristics, followed by four screening questions and the HELIA questionnaire, under the supervision of research assistants. The ethics committees of Mashhad and Shiraz Universities of Medical Sciences approved the study (Ethical codes: IR.MUMS.MEDICAL.REC.1401.702 and IR.SUMS.REC.1402.069, respectively).

###  Demographics

 Data collected included single-item questions for age, gender, city (Mashhad, Shiraz), job status (employed, unemployed (or housekeeper)), educational status (under the diploma, diploma, or higher), and participants’ status (inpatient, outpatient, or healthy).

###  Health Literacy Screening Questions

 SHLS is a four-item assessment tool which included Chew et al^14^ three screening items: “How often do you have someone (like a family member, friend, hospital/clinic worker or caregiver) help you read hospital materials?” (Help Read), “How often do you have problems learning about your medical condition because of difficulty understanding written information?” (Problems Reading), and “How confident are you filling out forms by yourself?” (Confident with Forms). These questions were translated into Persian. The forward-backward translation approach was used for translation. Accordingly, two independent English language professionals translated the English questionnaire into Persian. Afterwards, two professionals who had not received the original English questionnaire translated the Persian version back into English. A research team comprising clinical and language experts then compared it with the original version to identify any areas or inconsistencies where the meaning may have changed. The wording of the questions was slightly modified to accommodate language differences and enhance participants’ understanding. Also, we added the fourth question, “How well do you understand the medical prescriptions your doctor told you?” (Comprehension of Prescriptions), based on the experts’ opinions. Responses were scored on a Likert scale from 0 to 4. The response choices for “Help Read” and “Problems Reading” questions included always, often, sometimes, occasionally, and never, and for “Confident with Forms” and “Comprehension of Prescriptions” included not at all, a little bit, somewhat, quite a bit, and extremely. The total score ranges from 0 to 16 points, and higher scores represent a higher level of health literacy.

###  Validity and Reliability Assessment

 Content validity was assessed by a group of ten experts with backgrounds in epidemiology, biostatistics, medical education, community medicine, and public health, all have conducted research in the field of health literacy. The content validity index (CVI) was used to evaluate the screening questions’ relevancy based on a four-point scale (1 = not relevant, 2 = needs essential revision, 3 = relevant but needs revision, and 4 = very relevant). The CVI was calculated by dividing the number of respondents who scored three or four by the total number of them. A score below 0.70 indicates the item is unacceptable and should be removed.

 To assess the face validity, the questionnaire was given to relevant experts (with backgrounds in epidemiology, biostatistics, medical education, community medicine, and public health) for evaluation of each item for relevancy, clarity, difficulty, grammar, and vocabulary as a quick overall validity of the items, and they noted no specific problems.

 Internal consistency reliability was measured by Cronbach’s alpha, with an alpha value of 0.7 or higher considered acceptable. To evaluate the test-retest reliability, at least 40 participants completed the questionnaire twice within a two-week interval. Then, the Spearman-Brown correlation coefficient was computed. It was interpreted as follows: 0.40 to 0.69 represented a moderate correlation, 0.70 to 0.89 and 0.90 to 1 were considered strong and very strong correlations, respectively.

###  Comparison Standard

 To evaluate the performance of SHLS, we used two comparison standards: inadequate health literacy and the summation of inadequate and marginal as limited health literacy, as defined by the HELIA questionnaire. The HELIA, developed by Montazeri et al,^20^ is the first native questionnaire for the measurement of health literacy among the Iranian population. The psychometric properties of this instrument have been demonstrated in several studies.^21-23^ The HELIA is a 33-item reading assessment tool that contains five subscales (dimensions): reading (4 items), access to information (6 items), understanding (7 items), appraisal (4 items), and decision-making/behavioral intention (12 items). The total score ranges from 0 to 100 points. According to established cutoff scores, participants were divided into four categories: inadequate (0-50), marginal (50.1-66), adequate (66.1-84), and excellent health literacy (84.1-100). Also, two categories of inadequate and marginal were considered as limited (0-66), as well as adequate and excellent levels as sufficient health literacy (66.1-100).

###  Statistical Analysis

 The accuracy of SHLS was compared with two comparison standards: inadequate and limited health literacy, as assessed by HELIA. Receiver operating characteristic (ROC) curves, which illustrate the sensitivity versus (1-specificity), were used to determine the optimal trade-off between sensitivity and specificity compared with a reference standard. We calculated the areas under the receiver operating characteristic curve (AUROC) with 95% confidence intervals (CIs) to compare the overall performance of the screening items. The optimal screening of inadequate health literacy would have an AUROC of 1.0, while an AUROC below the null value of 0.5 indicates no information is provided. To determine which question(s) or different combinations of them would be optimal, we selected an individual question with the greatest AUROC and compared it with AUROCs of all other questions or combinations of them, considering the AUROC correlations from the same population. We calculated specificity, sensitivity, and negative and positive likelihood ratios (LRs) with 95% CI for different responses to screening questions. Youden’s J index ([sensitivity + specificity] - 1) was also calculated, which indicates overall effectiveness in terms of both sensitivity and specificity. Closer values to 1 indicate greater effectiveness. Furthermore, the Hanley test was used to compare the AUROCs. The effect size using Cohen’s d was also calculated for each question. Mann-Whitney U test was used for assessing the hypothesis of no difference across groups. We did not employ any imputation method in our analysis, since our dataset had no missing values. The *P* value < 0.05 was considered statistically significant. MedCalc version 22.009 (MedCalc Software, Mariakerke, Belgium) was used in this study.

## Results

###  Validity and Reliability Assessment

 The panel of experts included ten specialists evaluated face validity and content validity using CVI. For face validity assessment, experts’ opinions were collected and used to modify SHLS. None of the items were removed. The CVI scores for “Help Read”, “Problems Reading”, “Confident with Forms”, and “Comprehension of Prescriptions” were acceptable (0.92, 0.78, 0.85, and 0.85, respectively).

 Reliability was evaluated by calculating Cronbach’s alpha and the Spearman-Brown correlation coefficient. Cronbach’s alpha coefficients were more than 0.7 for all the factors (0.713), indicating that all items had acceptable internal consistency. The Spearman-Brown correlation coefficient was used to assess the test-retest reliability. It was calculated as 0.827, 0.928, 0.581, and 0.712 for the aforementioned screening questions, respectively (*P* value < 0.001 for all).

###  Participants’ Characteristics

 A total of 547 individuals were included in our study. Of them, 62% (339) were female, and most of them were between 18 and 45 years (415, 75.9%). The questionnaires were collected from outpatients (32.7%, 179), inpatients (32.5%, 178), and healthy individuals (34.7%, 190). The prevalence of inadequate and limited health literacy among study participants, based on the HELIA questionnaire, was 10.2% (56) and 35.1% (192), respectively (Table 1).

**Table 1:** Characteristics of study participants (N = 547)

**Variable**	**Subgroups**	**Frequency**	**Percent**
Age group	18-45	415	75.9
	46-65	132	24.1
Gender	Male	208	38.0
	Female	339	62.0
City	Shiraz	302	55.2
	Mashhad	245	44.8
Job-status	Employed	253	46.3
	Unemployed (or housekeeper)	294	53.7
Education	Under the diploma	123	22.5
	Diploma or higher	424	77.5
Participants status	Outpatient	179	32.7
	Inpatient	178	32.5
	Healthy	190	34.7
Health literacy level based on the HELIA questionnaire	Inadequate	56	10.2
	Marginal	136	24.9
	Adequate	234	42.8
	Excellent	121	22.1

HELIA: Health Literacy for Iranian Adults.

###  Detecting Inadequate and Limited Health Literacy

**Figure 1:**
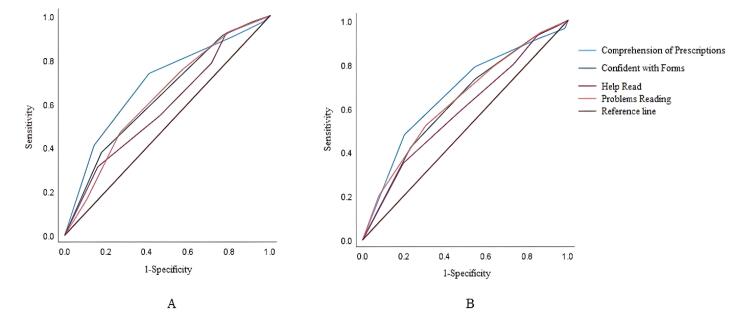
Receiver operating characteristic (ROC) curves of four screening questions to identify inadequate (A) and limited (B) health literacy

 The AUROCs with 95% CI for each of the screening questions to detect people with inadequate and limited health literacy are shown in Table 2. All the questions had statistically significant discriminatory ability. Compared to other screening questions, the “Comprehension of Prescriptions” item showed higher AUROC, with 0.690 (0.620-0.759) for inadequate health literacy and 0.666 (0.619-0.713) for limited health literacy. Figure 1 illustrates the ROC curves for each of the screening questions in detecting inadequate and limited health literacy. For both inadequate and limited health literacy, the Hanley test showed a significant difference in the AUROC between the “Help Read” and “Comprehension of Prescriptions” questions.

**Table 2:** Areas under the receiver operating characteristic (AUROC) and 95% confidence interval (CI) for each of the screening questions (N = 547)

**Screening questions**	**Inadequate health literacy**	**Limited health literacy **
Help Read	0.588 (0.511-0.666)	0.589 (0.540-0.639)
Problems Reading	0.629 (0.549-0.709)	0.635 (0.587-0.683)
Confident with Forms	0.635 (0.559-0.712)	0.627 (0.578-0.676)
Comprehension of Prescriptions	0.690 (0.620-0.759)	0.666 (0.619-0.713)

**Figure 2:**
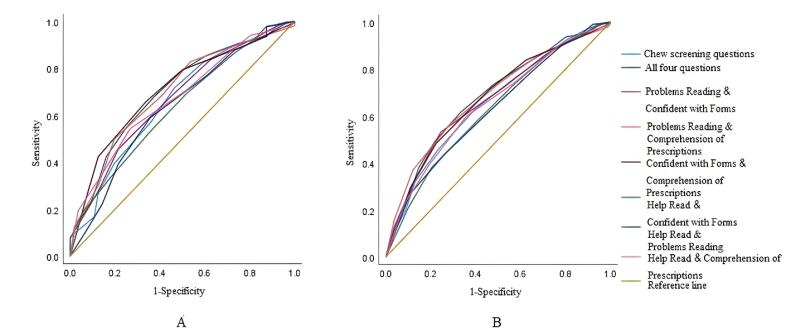
Receiver operating characteristic (ROC) curves of different combinations of screening questions to identify inadequate (A) and limited (B) health literacy

 To determine whether the combining of screening questions may enhance their performance, we performed analyses evaluating the screening performance of different combinations of questions. The AUROCs of various combinations of questions for detecting inadequate and limited health literacy are presented in Table 3. The AUROC of “Confident with Forms” & “Comprehension of Prescriptions” and all four questions were higher than the best-performing individual question (“Comprehension of Prescriptions”) for inadequate and limited health Literacy, respectively. Screening with “Confident with Forms” & “Comprehension of Prescriptions” had an AUROC of 0.705 (0.638-0.773) for inadequate health literacy, and screening with all four questions had an AUROC of 0.683 (0.636-0.729) for limited health literacy. The results indicated that these two combinations could alternatively be used as fair or reasonable screening tests. All the combinations had statistically significant discriminatory ability. ROC curves for different combinations of screening questions for identifying inadequate and limited health literacy are shown in Figure 2. For both inadequate and limited health literacy, based on the Hanley test, the comparisons of “Help Read & Problems Reading” with “All Four Questions,” “Confident with Forms & Comprehension of Prescriptions,” and “Problems Reading & Comprehension of Prescriptions” were significant. Similarly, the comparisons involving “Help Read & Confident with forms” with “All Four Questions,” “Confident with Forms & Comprehension of Prescriptions,” and “Problems Reading & Comprehension of Prescriptions” were also significant.

**Table 3:** Areas under the receiver operating characteristic (AUROC) and 95% confidence interval (CI) for different combinations of screening questions (N = 547)

**Screening questions**	**Inadequate health literacy**	**Limited health literacy**
All four questions	0.700 (0.629-0.771)	0.683 (0.636-0.729)
Chew screening questions	0.660 (0.584-0.737)	0.657 (0.610-0.704)
Help Read & Problems Reading	0.634 (0.555-0.713)	0.629 (0.581-0.677)
Help Read & Confident with Forms	0.634 (0.559-0.710)	0.629 (0.581-0.678)
Help Read & Comprehension of Prescriptions	0.670 (0.600-0.740)	0.651 (0.603-0.698)
Problems Reading & Confident with Forms	0.668 (0.594-0.742)	0.667 (0.621-0.714)
Confident with Forms & Comprehension of Prescriptions	0.705 (0.638-0.773)	0.674 (0.627-0.720)
Problems Reading & Comprehension of Prescriptions	0.695 (0.624-0.766)	0.682 (0.636-0.727)

 Sensitivities, specificities, Youden’s J indices, and negative and positive LRs with 95% CI for different responses of the screening questions are displayed in Tables 4 and 5. According to Youden’s J Index, “Somewhat” and “Quite a bit” were the optimal cutoff responses for detecting inadequate and limited health literacy, respectively. However, Youden’s J indices should be interpreted cautiously as the values for sensitivity and specificity are equally weighted. When using screening questions, sensitivity and specificity are not always regarded as equally important. It seems that sensitivity to be more valued than specificity.^24,25^ Therefore, we considered the response “Quite a bit” as the optimal screening threshold for “Comprehension of Prescriptions” to identify people with inadequate and limited health literacy. For inadequate health literacy, the LR + for this response was 1.45 (1.27-1.65), and the LR- was 0.35 (0.18-0.67). The response of “Quite a bit” or less confident increased the odds of inadequate health literacy by 1.45-fold, while a more confident response reduced the odds by 0.35-fold. The effect sizes for inadequate health literacy ranged from 0.340 for the “help read” question to 0.565 for the “comprehension of prescriptions” question. For limited health literacy, the LR + for this response was 1.53 (1.35-1.73), and the LR- was 0.42 (0.31-0.57). A response of “Quite a bit” or less confident increased the odds of limited health literacy by 1.53-fold, while a more confident response reduced the odds by 0.42-fold. The effect sizes for limited health literacy ranged from 0.323 for the “help read” question to 0.539 for the “comprehension of prescriptions” question. The Mann–Whitney U test results were significant for all questions in both the inadequate and limited health literacy groups. This indicated that, for both categories, question scores differed significantly from those with higher health literacy.

**Table 4:** Performance of screening questions for detecting inadequate health literacy (N = 547)

**Question**	**Criterion**	**Sensitivity**	**Specificity**	**Youden's J index**	**+LR (95% CI)**	**-LR (95% CI)**	Effect size* (95% CI)	***P*** value**
Help Read	≥ Never	100	0	0	1		0.340 (0.063, 0.618)	0.026
	≥ Occasionally	83.93	31.16	0.15	1.22 (1.07-1.39)	0.52 (0.28-0.95)		
	≥ Sometimes	53.57	54.58	0.08	1.18 (0.91-1.53)	0.85 (0.63-1.14)		
	≥ Often	28.57	78.41	0.06	1.32 (0.85-2.07)	0.91 (0.77-1.08)		
	≥ Always	21.43	92.06	0.13	2.7 (1.50-4.84)	0.85 (0.74-0.98)		
Problems Reading	≥ Never	100	0	0	1		0.484 (0.205, 0.763)	0.001
	≥ Occasionally	89.29	16.29	0.05	1.07 (0.97-1.18)	0.66 (0.30-1.44)		
	≥ Sometimes	73.21	46.64	0.19	1.37 (1.15- 1.64)	0.57 (0.37-0.89)		
	≥ Often	42.86	75.36	018	1.74 (1.24-2.44)	0.76 (0.60-0.96)		
	≥ Always	19.64	92.87	0.12	2.76 (1.48-5.11)	0.87 (0.76-0.99)		
Confident with Forms	≤ Extremely	100	0	0	1		0.533 (0.254, 0.812)	< 0.001
	≤ Quite a bit	82.14	37.68	0.19	1.32 (1.15-1.52)	0.47 (0.27-0.84)		
	≤ Somewhat	48.21	68.43	0.16	1.53 (1.13-2.06)	0.76 (0.58-0.98)		
	≤ A little bit	23.21	91.04	0.14	2.59 (1.49-4.51)	0.84 (0.73-0.98)		
	≤ Not at all	8.93	97.15	0.06	3.13 (1.17-8.37)	0.94 (0.86-1.02)		
Comprehension of Prescriptions	≤ Extremely	100	0	0	1		0.656 (0.376, 0.936)	< 0.001
	≤ Quite a bit	85.71	40.94	0.26	1.45 (1.27-1.65)	0.35 (0.18-0.67)		
	≤ Somewhat	58.93	73.73	0.32	2.24 (1.72-2.92)	0.56 (0.41-0.77)		
	≤ A little bit	17.86	90.63	0.08	1.91 (1.02-3.56)	0.91 (0.80-1.03)		
	≤ Not at all	3.57	97.15	0	1.25 (0.29-5.37)	0.99 (0.94-1.05)		

LR: likelihood ratio, CI: confidence interval, * Cohen’s d with inadequate category as the reference, ** Estimated using Mann-Whitney U test.

**Table 5:** Performance of screening questions for detecting limited health literacy (N = 547)

**Question**	**Criterion**	**Sensitivity**	**Specificity**	**Youden's J index**	**+LR (95% CI)**	**-LR (95% CI)**	Effect size* (95% CI)	***P*** value**
Help Read	≥ Never	100	0	0	1		0.323 (0.146, 0.500)	< 0.001
	≥ Occasionally	80.21	34.93	0.15	1.23 (1.11-1.37)	0.57 (0.41-0.78)		
	≥ Sometimes	53.65	57.75	0.11	1.27 (1.06-1.52)	0.8 (0.67-0.96)		
	≥ Often	26.56	80	0.06	1.33 (0.97-1.82)	0.92 (0.83-1.01)		
	≥ Always	14.58	93.52	0.08	2.25 (1.33-3.80)	0.91 (0.86-0.97)		
Problems Reading	≥ Never	100	0	0	1		0.498 (0.319, 0.676)	< 0.001
	≥ Occasionally	92.19	20	0.12	1.15 (1.08-1.23)	0.39 (0.23-0.66)		
	≥ Sometimes	69.27	52.11	0.21	1.45 (1.25-1.67)	0.59 (0.47-0.75)		
	≥ Often	36.46	78.87	0.15	1.73 (1.31-2.27)	0.81 (0.71-0.91)		
	≥ Always	13.54	94.37	0.07	2.4 (1.38-4.19)	0.92 (0.86-0.97)		
Confident with Forms	≤ Extremely	100	0	0	1		0.463 (0.285, 0.641)	< 0.001
	≤ Quite a bit	76.56	42.25	0.18	1.33 (1.18-1.49)	0.55 (0.42-0.74)		
	≤ Somewhat	45.31	73.24	0.18	1.69 (1.34-2.14)	0.75 (0.65-0.86)		
	≤ A little bit	16.15	92.68	0.08	2.2 (1.35-3.60)	0.9 (0.84-0.97)		
	≤ Not at all	5.21	97.46	0.02	2.05 (0.85-4.97)	0.97 (0.94-1.01)		
Comprehension of Prescriptions	≤ Extremely	100	0	0	1		0.539 (0.360, 0.718)	< 0.001
	≤ Quite a bit	79.69	47.89	0.27	1.53 (1.35-1.73)	0.42 (0.31-0.57)		
	≤ Somewhat	45.31	78.87	0.24	2.14 (1.66-2.77)	0.69 (0.60-0.80)		
	≤ A little bit	14.06	91.83	0.05	1.72 (1.05-2.82)	0.94 (0.88-1.00)		
	≤ Not at all	1.56	96.34	-2.1	0.43 (0.12-1.48)	1.02 (0.99-1.05)		

LR: likelihood ratio, CI: confidence interval, * Cohen’s d with inadequate category as the reference, ** Estimated using Mann-Whitney U test.

## Discussion

 Our study, the first to evaluate the psychometric properties of the Persian translation of the health literacy screening questions, showed that SHLS had acceptable psychometric properties. Chouinard et al^18^ evaluated the psychometric properties of French translation of Chew screening questions among patients with chronic disorders in a primary care setting. The intraclass correlation coefficient, assessing test-retest reliability, was 0.69 (0.45-0.83), and Cronbach’s alpha for internal consistency was 0.77. In another study, Cronbach’s alpha for Chew screening questions, administered by research assistants, was 0.79 and 0.71 among hospital and clinic patients, respectively.^7^ These screening questions have also been translated into Spanish,^16^ Arabic,^15^ and Turkish,^17^ but their test-retest reliability or internal consistency were not assessed. No data were found that evaluated the content validity and face validity of these health literacy screening questions.

 Our investigation is the first to validate the health literacy screening questions for identifying individuals with inadequate and limited health literacy using the HELIA questionnaire. Our study demonstrated that the “Comprehension of Prescriptions” question showed a better performance than the other three questions in detecting inadequate and limited health literacy. This question was slightly more accurate in identifying respondents with inadequate health literacy, as indicated by a higher AUROC than limited health literacy. The combination of “Confident with Forms & Comprehension of Prescriptions” had higher performance than the mentioned individual question for inadequate health literacy. Additionally, all four items together had a higher AUROC than “Comprehension of Prescriptions” for identifying limited health literacy, indicating that the tool as a whole is more sensitive than its components.

 Chew et al^13^ designed a 16-item self-reported health literacy screening questionnaire and then condensed it into a shortened version of three questions. They later revealed that the individual “Confidence with Forms” question was the best predictive of inadequate health literacy out of three questions. However, this item performed less well in those with inadequate/marginal health literacy.^14^ Other studies have also shown that it performed best in detecting inadequate health literacy.^16,26-28^ However, in a sample of patients at a university-based vascular surgery clinic^29^ and those in a Veterans Affairs (VA) preoperative clinic,^13^ the question “Help Read” showed better performance in detecting inadequate health literacy. In contrast to our results, Chew et al^14^ found that no combination of three questions had better performance in comparison to the “Confidence with Forms” question. These different results may be related to factors that have not yet been clarified, including the demographics of study populations and the context of healthcare systems. It is important to conduct further research to understand the reasons behind these differences. In another study evaluating the performance of Chew screening questionnaire among English and Spanish-speaking individuals with type 2 diabetes, it has been shown that the summative scale performed as well as the single question “Confident with Forms”.^28^ Haun et al^11^ investigated the efficacy of these questions along with the fourth question “How often do you have a problem understanding what is told to you about your medical condition?” to assess difficulties with auditory health information in a VA ambulatory care setting. Consistent with our findings, the combination of all questions had a greater AUROC than either of the individual questions for identifying inadequate and inadequate/marginal health literacy.

 To establish the optimal cut point for a screening instrument, several factors need to be considered, including test accuracy, testing expenses, the prevalence of low health literacy, and the benefits of detecting true positives and false positives. If a screening test is intended to identify individuals lacking adequate health literacy skills, one would select a cutoff response with high sensitivity and low LR- thereby those with negative test results are most probably to have adequate health literacy. But if the aim is to detect people with poor health literacy, a cutoff response with high specificity and high LR + would be used thereby those with positive test results are most probably to have poor health literacy. However, based on the prevalence of insufficient health literacy in the study population, a positive or negative test could have different implications.^14^ We recommended using the “Quite a bit” response to “How well do you understand the medical prescriptions your doctor told you?” as the optimal cutoff value for detecting inadequate and limited health literacy. This cut-point, with reasonable specificity, identified 79.69% of individuals with limited and 85.71% of people with inadequate health literacy in our study population. The optimal cutoff values for these questions need to be determined in other clinical settings with different prevalence of low health literacy^13^ and depending on whether specificity or sensitivity should be emphasized.^27^

 To achieve successful health-related outcomes, health literacy assessment must be incorporated into clinical settings.^30^ The SHLS developed in this study can be used in primary care settings, where health care professionals often face challenges in establishing an effective relationship with patients from diverse educational and cultural backgrounds. It can also be useful for the management of patients with chronic disorders to improve their compliance and prognosis. As a validated, easy-to-use screening tool, SHLS can help healthcare providers identify individuals with limited health literacy who may need targeted support and communication strategies. SHLS can also be implemented in electronic medical record systems and serve as a helpful guide for health care providers when encountering patients, especially in busy clinical settings.

 Our research featured a number of notable strengths. We chose a random sample of people from two of the biggest cities in the east and center of Iran with different cultural context. In addition, study participants were selected from inpatient and outpatient settings with different socioeconomic status as well as healthy people to achieve a more representative results. Due to the large sample size, we were able to measure the effectiveness of screening questions much more precisely. We also added a fourth question regarding the comprehension of medical prescriptions to better adaptation to our healthcare system context.

 In relation to literacy levels, cultural beliefs regarding health and illness have a significant effect on the individuals’ ability to comprehend and adhere to a physician’s advises.^31^ It is argued that cultural minority patients are more affected by low health literacy than those from the dominant culture, due to interactions between literacy, language barriers, and biased experiences.^32^ Moreover, cultural beliefs affect educational levels and the ability to obtain healthcare information.^30^ The extent of cultural diversity among various populations and the affected factors may contribute to disparities in health literacy levels and assessment tool performances across different studies. The main advantage of the SHLS is that it is brief, requiring only a few minutes to complete, and is a useful tool for screening limited health literacy among Iranian populations. Given the cultural diversity in our country and its impact on health literacy, the SHLS can be utilized as a screening tool, and based on its results, more culture-based methods can be used to improve the quality of health care services.

 Despite these strengths, this study has some drawbacks. As a limitation for the screening tool, the limited number of questions in SHLS (four items), make it difficult to conduct factor analysis. Demographic characteristics including age and educational status were not evaluated alone or combined with SHLS to identify people with inadequate and limited health literacy. We used self-reported or subjective questionnaire in this study and response bias remains a possibility. While we incorporated participants from three distinct groups in two of Iran’s main cities, larger multicentric studies are necessary to enhance the generalizability for the entire Iranian population.

 Future research could focus on how screening results be used for improving physician-patient communication and adding calculators, demographic variables, and other tools to address low health literacy. The effectiveness of separate ways of administrating screening questions (written or verbal) could also be explored. People with positive screening for low health literacy may benefit from interventions using special support and communication methods to help them navigate the healthcare system effectively.

## Conclusion

 Considering our results, the single question “How well do you understand the medical prescriptions your doctor told you?” and the summative scale of four screening questions as SHLS could discriminate people with inadequate and limited health literacy. Our study suggests the application and further evaluation of SHLS in epidemiological and clinical research among different populations.

## Competing Interests

 None to declare.

## Ethical Approval

 The ethics committees of Mashhad and Shiraz Universities of Medical Sciences approved this study (Ethical codes: IR.MUMS.MEDICAL.REC.1401.702 and IR.SUMS.REC.1402.069, respectively). Informed verbal consent was obtained from study participants before completing the questionnaire.
